# Photo-Inactivation of *Staphylococcus aureus* by Diaryl-Porphyrins

**DOI:** 10.3390/antibiotics12020228

**Published:** 2023-01-20

**Authors:** Viviana Teresa Orlandi, Eleonora Martegani, Nicola Trivellin, Fabrizio Bolognese, Enrico Caruso

**Affiliations:** 1 Department of Biotechnologies and Life Sciences, University of Insubria, Via J.H. Dunant 3, 21100 Varese, Italy; 2 Department of Industrial Engineering, University of Padova, Via Gradenigo 6A, 35131 Padova, Italy

**Keywords:** diaryl-porphyrins, Photodynamic Antimicrobial Chemotherapy (PACT), *Staphylococcus aureus*

## Abstract

Photodynamic Antimicrobial Chemotherapy (PACT) has received great attention in recent years since it is an effective and promising modality for the treatment of human oral and skin infections with the advantage of bypassing pathogens’ resistance to antimicrobials. Moreover, PACT applications demonstrated a certain activity in the inhibition and eradication of biofilms, overcoming the well-known tolerance of sessile communities to antimicrobial agents. In this study, 13 diaryl-porphyrins (mono-, di-cationic, and non-ionic) **P1**–**P13** were investigated for their potential as photosensitizer anti-*Staphylococcus aureus*. The efficacy of the diaryl-porphyrins was evaluated through photo-inactivation tests. Crystal-violet staining combined with viable count techniques were aimed at assaying their anti-biofilm activity. Among the tested compounds, the neutral photosensitizer **P4** was better than the cationic ones, irrespective of their corresponding binding rates. In particular, **P4** was active in inhibiting the biofilm formation and in impairing the viability of the adherent and planktonic populations of a 24 h old biofilm. The inhibitory activity was also efficient against a methicillin resistant *S. aureus* strain. In conclusion, the diaryl-porphyrin family represents a reservoir of promising compounds for photodynamic applications against the pathogen *S. aureus* and in preventing the formation of biofilms that cause many infections to become chronic.

## 1. Introduction

*Staphylococcus aureus* permanently colonizes the nose of 30% of individuals, while most of the population are intermittent carriers. An important issue occurs in fragile patients when *S. aureus* contaminates skin wounds and mucous membranes reaching other deeper tissues: infections rapidly develop causing a variety of acute and chronic diseases with increasing severity. Thanks to a great arsenal of virulence factors and toxins, *S. aureus* causes toxic shock syndrome, scalded skin syndrome, food poisoning, endocarditis, and haemolytic pneumonia [1,2]. In addition, the patients with compromised immune systems, those frequently subjected to injections, or those who are permanently catheterized acquire *S. aureus* more easily in nosocomial environments. Indeed, the biofilm formation on tissues or clinical devices favors *S. aureus* to establish the infection and elude the host immune response [3]. In clinical history, *S. aureus* was the first microorganism to be shown growing in a biofilm form on the surface of an endocardial pacemaker and causing a persistent bacteraemia [4]. Nowadays, biofilm formation by *S. aureus* is a current problem in infection care, prevalently causing medical device-associated infections on heart valves, catheters, and joint prosthetics, and is increasingly tolerant to host immune responses and antibiotic treatments. The initial *S. aureus* attachment on the host or inert surfaces can occur through electrostatic interactions or be mediated by microbial surface components that recognize fibrinogen, fibronectin, and collagen. Subsequently, biofilm develops as a mass of confluent cells in a proteinaceous matrix, also containing eDNA. The formation of three-dimensional microcolonies leads to the production of a robust mature biofilm. In the last phase of the biofilm life cycle, planktonic cells can be dispersed to initiate biofilm formation on other sites [5].

In addition to biofilm, the control of *S. aureus* infection becomes difficult because of the arising resistant strains. Indeed, this superbug is able to share the genetic determinants of antimicrobial resistance by an efficient horizontal gene transfer [6]. The first evidence of methicillin-resistant *Staphylococcus aureus* (MRSA) was reported in the 1960s and, in recent years, MRSA strains have a prevalence of 25–50% within *S. aureus* isolates [7]. For all the mentioned reasons, *S. aureus* deserves much attention in the clinical environment and represents one of the most virulent and antimicrobial resistant bacterial species together with *Enterococcus* spp., *Klebsiella pneumoniae*, *Acinetobacter baumannii*, *Pseudomonas aeruginosa*, and *Enterobacter* spp. For these, the term ESKAPE group was created [8].

In the era of antimicrobial resistance, emerging strategies are under investigation to combat ESKAPE pathogens. Among these, the following ones can be highlighted: antimicrobial peptides, bacteriophages, nanomaterials, cold plasma, and light-based techniques [9,10]. In particular, the use of visible light alone or combined with drugs is very promising. Indeed, Photodynamic Antimicrobial Chemotherapy (PACT) showed to be efficient against *S. aureus*. This technique is based on the photo-oxidative stress induced by the combination of dyes (PSs; photosensitizers) with visible light in the presence of oxygen. When the arisen burst overcomes the natural defense of cells against reactive oxygen species (ROS), the bactericidal effect is obtained. Acridine orange has been the first dye used as PS. The currently available PSs are usually conjugated unsaturated organic molecules belonging to different chemical families: tetrapyrrolic-based compounds such as porphyrins, chlorins, bacteriochlorins and phthalocyanines, phenothiazines (methylene blue and toluidine blue), rose bengal, fullerenes, and boron-dipyrromethenes (BODIPYs). Furthermore, many natural substances demonstrated to act as PS molecules, such as chlorophyll, curcumin, riboflavin, phenalenone, xanthene, and their derivatives [11,12,13].

An ideal antimicrobial PS should be selectively active against microbial cells upon its photoactivation and safe for human tissues. No toxicity and mutagenicity in the dark should be present toward both eukaryotic and prokaryotic cells [14]. In addition, the low-molecular weight of PS compounds seems to facilitate the penetration in microbial biofilms. A high singlet oxygen quantum yield is a desired feature to elicit the production of reactive oxygen species [15]. The absorption coefficient should be appropriate for an effective penetration of light in the infection site. In particular, short wavelengths (440 nm) could be used for skin infections, while long ones are preferable for penetration in deeper human tissues (540–650 nm) [16]. The effect of light on the PS molecule, known as photobleaching, should be considered: for therapeutic purposes, the photobleaching effect after the treatment could be suitable to avoid tissue photosensitivity, while for surface disinfection purposes a very low photobleaching effect is preferable, to reach an extended photosensitizing effect [17].

The Gram-positive bacterial species such as *S. aureus* showed to be sensitive to different PSs, irrespective of charge [18,19]. Their thick cell wall with a widespread anionic charge conferred by the presence of lipoteichoic acid seems responsible for a self-promoted uptake of neutral, cationic, and anionic PSs [20]. In this study, we evaluated the efficacy of a panel of synthetic non-ionic and cationic diaryl-porphyrins, previously tested against *Pseudomonas aeruginosa* and *Candida albicans* [21,22], in photo-inactivating *S. aureus*.

## 2. Results

In this study, a panel of non-ionic, monocationic, and dicationic diaryl-porphyrins (Table 1) was tested to find potential PS/s against *Staphylococcus aureus*. The compounds were previously described [23,24,25]

### 2.1. Effect of Diaryl Porphyrins on Viability of Staphylococcus aureus

**Figure 1:**
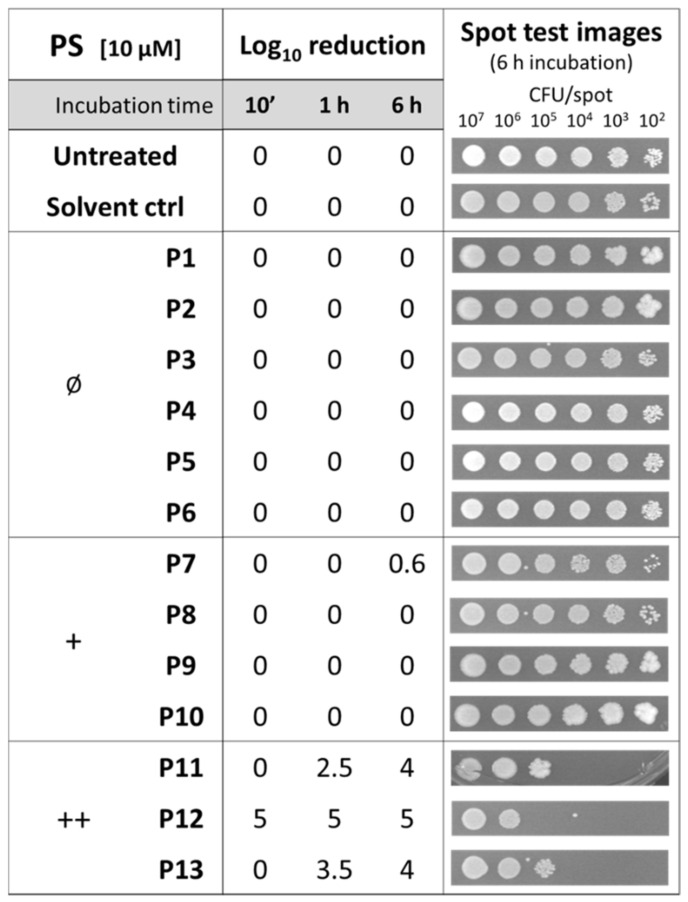
Analysis of intrinsic toxicity of diaryl-porphyrins (**P1**–**P13**) administered at a concentration of 10 µM to *Staphylococcus aureus* ATCC 6538P for 10 min, 1 h, and 6 h of dark incubation. Untreated and DMSO-treated samples (“Solvent ctrl”) were included as controls. Log_10_ reduction values represent the mean of at least three independent experiments. Representative images reported in the last column refer to growth spots at decreasing bacterial densities (from 10^7^ to 10^2^ CFU/spot) upon 6h of dark incubation with the corresponding PS.

Since no intrinsic toxicity should be associated with an ideal PS, the activity of the diaryl-porphyrins was investigated on *S. aureus*. The effect of the DMSO solvent was also included as a control. The spot-test method allowed for an assay of the effect of PSs administered at the same concentration (10 µM) to different samples with decreasing cell concentrations and after different times of dark incubation: 10 min, 1 h, and 6 h, respectively (Figure 1).

**Figure 2:**
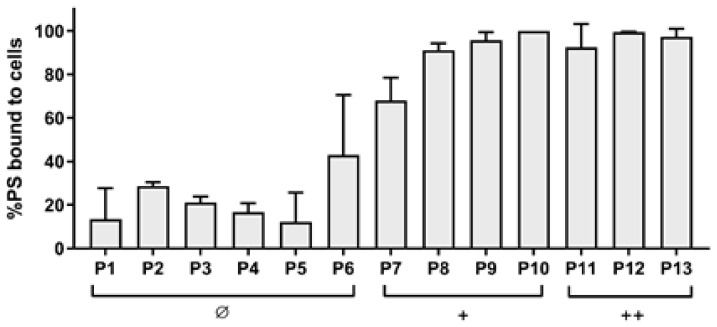
Binding assay of diaryl-porphyrins (**P1**–**P13**) to *S. aureus* ATCC 6538P. Values are presented as a percentage of PS bound to cells upon 1 h of dark incubation. The neutral (Ø), monocationic (+), and dicationic (++) porphyrins were administered at a concentration of 10 μM. The experiments were performed in triplicate and standard deviation is reported.

The dicationic compounds were intrinsically toxic: the antimicrobial activity of **P12** was independent from the incubation time, while that of **P11** and **P13** increased with longer incubation times. To evaluate if those compounds were more able than the others to interact with cells, the binding rates were evaluated. As shown in Figure 2, even if the cationic charge causes the interaction with bacteria compared with the neutral ones to be easier, no difference was observed between mono- and dicationic porphyrins. Furthermore, no statistically significant difference of the binding rate was observed among the compounds belonging to the same group, neutral, monocationic, and dicationic ones, respectively.

### 2.2. Photodynamic Activity of Diaryl-Porphyrins

**Figure 3:**
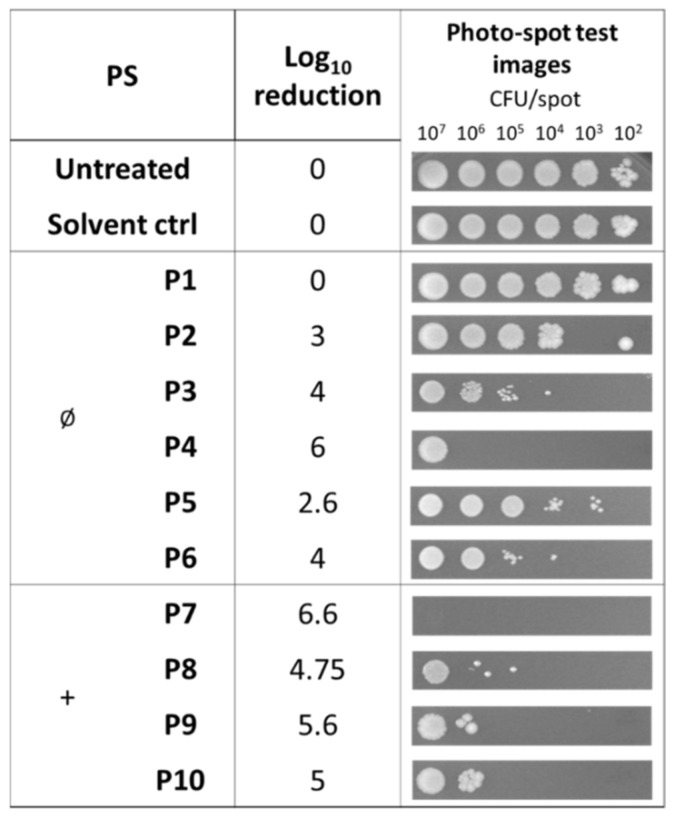
Photodynamic activity of diaryl-porphyrins on *S. aureus* ATCC 6538P evaluated by the photo-spot test. The neutral (Ø) and monocationic (+) porphyrins were administered at a final concentration of 10 μM. “Untreated” lane corresponds to irradiated cells without PSs or DMSO. “Solvent control” corresponds to cells irradiated in the presence of DMSO. After 1 h of dark incubation and irradiation under 410 nm light (20 J/cm^2^), the cells were incubated at 37 °C O/N. Growth spots were checked and representative images are reported in the last column. Log_10_ reduction values represent the mean of at least three independent experiments.

In this study, we searched for compounds that kill bacteria only upon irradiation. For this reason, the photoactivity was evaluated for those compounds that did not show any intrinsic toxicity (Figure 3). The non-ionic compound **P4** caused a clear reduction in the cell viability of samples at 10^6^ cells/spot and the cationic **P8**–**P10** were able to inactivate samples of bacteria with a lower density of 10^5^ cells/spot. In this experimental setup, porphyrin **P7** showed the best killing rate (6.6 Log unit reduction).

**Figure 4:**
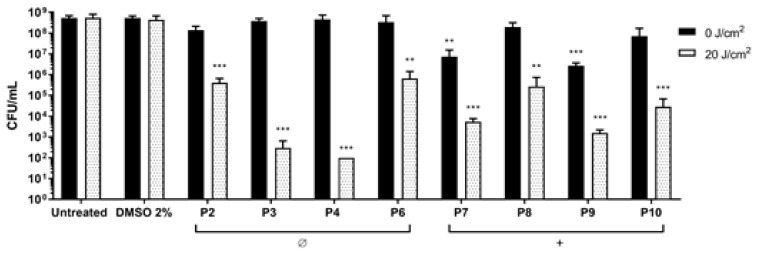
Photodynamic inactivation of *S. aureus* ATCC 6538P. After 1 h of dark incubation with **P2**–**4, P6,** and **P7**–**P10** at 10 µM concentration, cells were irradiated under light at 410 nm at 20 J/cm^2^ (grey bars) or kept in the dark (black bars). Values, presented as CFU/mL, are the mean of at least three independent experiments and the bars represent standard deviations. Statistical analyses using one-way ANOVA were performed between treated samples and reference untreated control (** *p* < 0.01; *** *p* < 0.0001).

*S. aureus* was treated with non-ionic (**P2**–**P6**) and monocationic porphyrins (**P7**–**P10**) (10 µM final concentration) and blue light was delivered at 20 J/cm^2^ (Figure 4). Non-ionic compounds were non-toxic in dark conditions, while upon irradiation they caused significant reductions: **P2** and **P6** caused a reduction of 2 Log units in the cell viability, while **P3** and **P4** showed the best PDT effect, causing reductions of 5 and 6 Log units, respectively, reaching the detection limit of the method (10^2^ CFU/mL). In this setup, the two monocationic PSs **P7** and **P9** showed intrinsic toxicity in the dark and were discarded from further analyses. Upon irradiation, **P8** and **P10** caused a decrease of 2 and 3 Log units, respectively.

### 2.3. Antibiofilm Activity of Porphyrins against S. aureus

**Figure 5:**
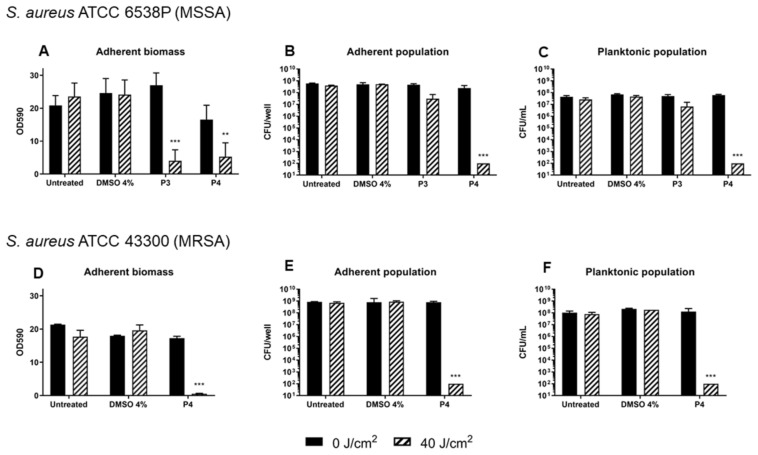
Inhibition of biofilm formation of *S. aureus* ATCC 6538P (**A**–**C**) and ATCC 43300 (**D**–**F**) upon photodynamic treatment with diaryl-porphyrin **P3** and **P4** (20 μM). Porphyrins were activated by 410 nm radiation at 40 J/cm^2^. The graphs report values of the optical density at 590 nm (OD 590) after biofilm staining with crystal violet (**A**,**D**), values of adherent population density (CFU/well) (**B**,**E**), and planktonic population concentration (CFU/mL) (**C**,**F**). Dark control samples are represented as black bars and light-treated samples as striped bars. Values represent the mean of at least three independent experiments ± the standard deviation. Statistical analyses using one-way ANOVA were performed between treated samples and reference untreated control (** *p* < 0.01; *** *p* < 0.0001).

The preliminary screening of diaryl-porphyrins activity on *S. aureus* cells revealed that two non-ionic molecules, **P3** and **P4**, had the best antimicrobial effect. Thus, these compounds were tested to evaluate their potential in inhibiting the biofilm formation. *S. aureus* was irradiated under blue light at 40 J/cm^2^ or treated with **P3** and **P4** 20 µM to rule out any possible toxicity of light or PSs, respectively. After 24 h of biofilm growth, the adherent biomass and viability of sessile and planktonic cells were determined (Figure 5A–C). The total biomass (OD590) and the viability of the adherent and planktonic phases did not change significantly upon treatment with DMSO in the dark or after irradiation compared to the untreated cells. Furthermore, irradiation alone did not show anti-biofilm activity. In the dark, **P3** did not influence the biofilm formation and the slight decrease in adherent biomass caused by **P4** was not statistically significant. Interestingly, even if both PSs caused a great decrease in total biomass upon light activation, their influence on cell viability was greatly different: **P3** was inefficient in inhibiting the growth of both planktonic and adherent phases, except for a slight decrease of ~1 Log unit compared to the dark control. On the other hand, the photoactivation of **P4** reduced both adherent and planktonic populations of ~6 Log units compared to the dark control, inhibiting the biofilm formation. To sum up, porphyrin **P3** seemed to have an inhibitory effect on biofilm matrix production but not on cell viability, while porphyrin **P4** showed the best anti-biofilm performances on the MSSA strain. Thus, **P4** was administered to an MRSA strain (Figure 5D–F). The amount of biofilm produced by the MRSA strain, evaluated as adherent biomass and bacterial concentrations (adherent and planktonic phases), was comparable to that of MSSA. Furthermore, the photodynamic treatment with porphyrin **P4** was successful as in MSSA: a significant decrease in the total biomass of MRSA and a killing effect of ~6 Log units both on adherent and planktonic cells was observed. Thus, the diaryl-porphyrin **P4** showed good photo-inactivating properties against *S. aureus*, independently from the *S. aureus* antibiotic susceptibility profile.

### 2.4. Photodynamic Eradication of Formed Biofilm

**Figure 6:**
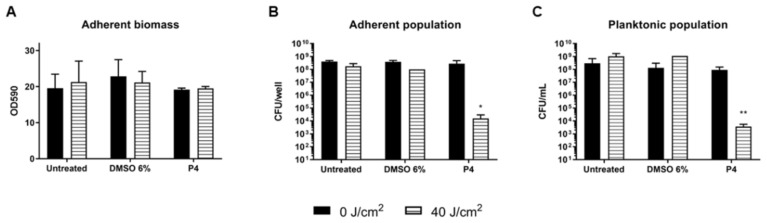
Assay of eradication of *S. aureus* ATCC 6538P biofilm by porphyrin **P4** (30 µM) upon irradiation with light at 410 nm (40 J/cm^2^). Adherent biomass of biofilm upon PDT is represented as OD590 (**A**) and cell viability is expressed as CFU/well for adherent population (**B**) and as CFU/mL for planktonic biomass (**C**). Values represent the mean of at least three independent experiments ± the standard deviation. Statistical analyses using one-way ANOVA were performed between treated samples and reference untreated control (* *p* < 0.05; ** *p* < 0.01).

The eradication of 24 h *S. aureus* biofilm using **P4** porphyrin (30 µM) PDT treatment was also assayed (Figure 6). The irradiation with light at 410 nm (40 J/cm^2^) and the administration of DMSO 6% *v*/*v* both in dark or under irradiation did not affect neither the total biomass nor the cell viability. Additionally, **P4** administration in dark or light conditions did not affect the total biomass of the biofilm, suggesting that no impairment of the biofilm matrix happened during PACT. The antimicrobial effects were instead observed when the cell viability was measured upon porphyrin and light exposure: a significant reduction of ~5 and 4 Log units was observed for the adherent and planktonic populations, respectively (Figure 6B,C).

## 3. Discussion

Porphyrin compounds present many chemico-physical features that cause them to be good photo-antimicrobials to apply in the clinical field. In the present era of drug-resistant bugs, the great potential of porphyrins has been supported by both in vitro and in vivo approaches [26,27]. If tetraaryl-porphyrins, differently substituted in *meso*-positions, have been widely investigated in PACT [28,29,30], fewer papers report the potential of 5,15-substituted diaryl-porphyrins. In particular, Burda and colleagues found that diaryl-porphyrins had enhanced potency in the inactivation of *S. aureus* compared to the related tetra-*meso*-substituted compounds [31]. In addition, the photodynamic inactivation of *C. albicans* yielded better results with a dicationic diaryl-porphyrin compared to the commercial 5,10,15,20-tetrakis(1-methyl-4-pyridyl)-21H,23H-porphine tetra-p-tosylate salt (TMPyP) [32]. Recently, a study tested a panel of diaryl-porphyrins as potential PSs to photo-inactivate *P. aeruginosa* and *C. albicans*, respectively [21,22]. In this study, *S. aureus* was considered as a Gram-positive model to be treated with the same panel of compounds and, thus, complete the screening.

When searching for antimicrobial photosensitizers in sensu stricto, i.e., compounds active only upon irradiation, any intrinsic toxicity should be avoided. The diaryl-porphyrins considered in this study bear ionic and hydrophobic groups differently disposed on the two appendages in 5 and 15 positions, building a panel of symmetric or asymmetric compounds with non-ionic or positive total charges. In dark conditions, the dicationic symmetric **P11**–**P13** caused a certain killing rate of *S. aureus* and for this reason they were not considered as ideal photosensitizers. On the other hand, the asymmetric monocationic diaryl-porphyrins were not intrinsically toxic and, since dicationic and monocationic diarylic-porphyrins showed very similar binding rates, this parameter seems not predictive of intrinsic toxicity in *S. aureus*. In general, cationic molecules bind the carboxylate groups of proteins, anionic residual of peptidoglycan, phosphate groups of lipoteichoic, and teichoic acids. Thus, the dicationic compounds could modify the cell envelope and increase cellular permeability compromising the physiological balance in a more disruptive manner than monocationic porphyrins [33]. Among dicationic compounds, **P11** and **P13** increased their antimicrobial effect after increasing dark incubation time, while **P12** was the most toxic even after a short incubation time (10 min). Thus, the degree of toxicity seems to be unrelated to the length of arylic chains on tetrapyrrolic nucleus: **P11** and **P13** bear the shortest and the longest aryl chain, respectively. However, in antimicrobial fields, these compounds deserve attention, especially **P11**. This porphyrin was intrinsically toxic to *S. aureus* and not to *P. aeruginosa*. The observed selectivity seems not related to a differential **P11** binding rate, as it tightly binds both Gram-positive and Gram-negative bacterial cell types [21]. The action mechanism of **P11** should be further investigated, especially if a specific microbial target could be found at the cell-envelope or at the cytoplasmic level. Indeed, narrow spectrum antimicrobials are preferred to broad-spectrum ones in order to avoid the uncontrolled diffusion of resistance genes, if any could be involved in **P11** activity [34].

The neutral diaryl-porphyrins did not show any intrinsic toxicity in *S. aureus* and their binding efficiency (12–40%) was lower than that shown by the cationic diaryl-porphyrins (70–100%). Still, *S. aureus* was more prone to bind neutral diaryl-porphyrins than *P. aeruginosa* [21]. In accordance with the literature, the porous cell wall of Gram-positive bacteria likely allows for the passage of neutral PSs, but differently from Gram-negative bacteria. For example, non-ionic PSs such as tetra-pyrrole based compounds, curcumin, and hypericin were efficient in the photokilling of Gram-positive, but not of Gram-negative, bacteria [35].

Most neutral and mono-cationic diaryl-porphyrins were active in a photo-spot test against *S. aureus*. However, non-ionic diaryl-porphyrins showed to be more suitable PSs for the photo-inactivation of *S. aureus* cells than cationic ones. On the other hand, monocationic and dicationic diaryl-porphyrins showed to be good PSs for the treatment of *P. aeruginosa*. These results highlighted that different diaryl-porphyrins could be used to inactivate different pathogens in a tailored PACT approach.

As a matter of fact, the best PACT performances were displayed by non-ionic porphyrins **P3** and **P4** that only differ for a C4 bromoalkyl chain in the *meso*-position. Since both compounds showed <20% of PS binding efficiency with *S. aureus* cells, a low threshold of binding rate is enough for *S. aureus* inactivation. However, when tested on biofilms, **P3** was not as efficient as **P4** in the inhibition of *S. aureus* biofilm formation. While **P3** prevented the formation of extracellular matrix biofilm without impairing the cellular viability of planktonic and sessile populations, **P4** was successful in inhibiting both matrix and cellular components. The biofilm formation in *S. aureus* is regulated by a complex and intricate network of factors that combine physiological processes in response to environmental and host stimuli. The most prominent regulation system of *S. aureus* is mediated by the accessory gene regulator (*agr*) Quorum Sensing (QS) system activated by an autoinducing peptide (AIP). The related up- and down-regulation of specific genes enhances biofilm formation and the production of virulence factors [36,37]. Thus, **P3** could interfere with QS components of biofilm machinery and prevent matrix production without impairing cell viability. **P4** seems to impair cell viability at the inoculum level, thus preventing the formation of a biofilm community even after 24 h of incubation. The rate of biofilm inhibition is higher than that recently obtained with curcumin on methicillin-resistant *S. aureus* [38]. Furthermore, **P4** was also active in inhibiting the biofilm formation of a methicillin-resistant strain. This result is an added value, especially for nosocomial infections caused by drug-resistant strains. The eradication of a formed biofilm is more difficult than the inhibition of biofilm formation. Beirao et al. achieved the goal through the photodynamic treatment of *S. aureus* biofilm by 20 µM tetracationic porphyrin TMP [39]. Moreover, TAPP porphyrin at 20 µM was active on *S. aureus* biofilm, causing a biomass depletion of 3 Log units [40]. The neutral diaryl-porphyrin **P4** at 20 µM concentration reached a good killing effect in adherent and planktonic populations of *S. aureus* biofilms. These results support diaryl-porphyrins PACT as an appropriate and preventive disinfection approach for inert surfaces of medical devices. Furthermore, PACT could be also applicable to localized superficial infections in oral cavities or on the skin, to avoid the beginning of chronic infections and the spread of microbial pathogens in other body districts.

The current in vitro screening performed on *S. aureus* and the previous one on *P. aeruginosa* identified new diaryl-porphyrins as promising next-generation antimicrobials. As skin infections can be easily treated, the chosen diaryl-porphyrins should be tested in vivo in animal models.

## 4. Materials and Methods

### 4.1. Photosensitizers

The previously described panel of 13 diaryl-porphyrins [21,22,23,24,25] has been used in this study (Table 1): **P1**–**P6** porphyrins are neutral molecules, while **P7**–**P10** are mono-cationic and **P11**–**P13** di-cationic, respectively. The PSs were dissolved in DMSO at a final concentration of 1 or 0.5 mM, as requested, and stored at 4 °C until needed.

### 4.2. Microbial Strains and Culture Conditions

Two strains of *S. aureus* were used, ATCC 6538P (MSSA) and ATCC 43300 (MRSA), respectively. The cells were grown overnight in a Tryptic Soy Broth (TSB) medium at 37 °C on an orbital shaker at 200 rpm or in solid media (15 g/L agar) at 37 °C.

### 4.3. Light Source

The lighting unit device is equipped with a head composed by 25 high power LEDs with maximum emission peak at 410 nm light, suitable for the activation of porphyrins and allows for the uniform irradiation of a square area of 75 mm × 75 mm. The system is powered by a specific PC based control system, which allows the setting of irradiation time and irradiance values for a precise setting of the radiation fluence rate.

### 4.4. Photo-Spot Test

The spot test previously optimized [21,41] was used to screen the intrinsic toxicity and the photoactivity of diaryl-porphyrins. Upon overnight growth in TSB, the *S. aureus* cultures were suspended in phosphate-buffered saline (PBS-KH_2_PO_4_/K_2_HPO_4_ 10 mM, pH 7.4) and 10-fold serially diluted from ~10^9^ to ~10^4^ CFU/mL in 96-well plates. The PSs (10 µM final concentration) were administered to bacterial samples. The untreated samples and DMSO treated samples were included as controls.

To investigate the intrinsic toxicity of PSs, bacterial suspensions were incubated in the dark, at increasing times, to avoid interference by the undesired photoactivation of PSs and to permit the interaction between porphyrins and cells. After 10 min, for 1 h or 6 h of dark incubation, respectively, volumes of ~5 µL of each sample were replica plated on LB agar. After overnight incubation at 37 °C, the growths of the treated samples were compared to the control spots of decreasing cell density (from ~10^7^ to ~10^2^ CFU/spot, respectively). The spot tests were performed at least in triplicate.

To investigate the photo-inactivation rates of diaryl-porphyrins, volumes of ~5 µL of bacterial suspensions obtained as previously described were replica plated on LB agar and irradiated under 410 nm light at a fluence rate of 20 J/cm^2^ (100 mW/cm^2^, 200 s). After overnight incubation at 37 °C, the spot growth was checked and compared to an untreated control. For example, if growth was not observed at a 10^2^ CFU/spot, a 2 Log unit decrease was recorded. Thus, higher values of growth reduction correspond to higher antimicrobial efficiency. The photo-spot tests were performed at least in triplicate.

### 4.5. Photo-Inactivation of Suspended Cells

Upon overnight growth, *S. aureus* cells (~10^9^ CFU/mL) were ten-fold diluted in PBS, to reach approximate concentrations of 10^8^ CFU/mL. The porphyrins were added to cell suspensions at a final concentration of 10 µM. Untreated cells, DMSO-treated cells, and no irradiated controls were also included. The cells were incubated in the dark for 60 min at 37 °C and then irradiated (20 J/cm^2^). Soon after irradiation, the number of viable cells was evaluated by viability count, plating 10 µL of serial dilutions on LB Agar. After overnight incubation at 37 °C, the corresponding cellular concentration was expressed as CFU/mL. The experiments were performed at least in triplicate.

### 4.6. Photosensitizer Binding Assay

All the PSs were tested for their ability to bind bacterial cells. Overnight cultures of *S. aureus* ATCC 6538P were centrifuged at 5000× *g* for 10 min and the supernatants were removed. The pellets were suspended and 10-fold diluted in PBS. The porphyrins were added to the cells at the concentration of 10 µM and incubated for 1 h at 37 °C in the dark. The untreated cells, PS-treated cells, and cells added with DMSO 4% (*v*/*v*) were included as controls. To rule out the intrinsic toxicity of PSs, the cell viability was assessed for all the samples upon dark incubation by the previously explained colony count method. After dark incubation, the samples were centrifuged (10,000× *g* for 5 min) and the supernatants were transferred in 2 mL cuvettes (Sarstedt^TM^). The absorption spectra were recorded (k = 380–700 nm) using a Varian Cary 50 UV-VIS Spectrophotometer. A calibration plot (µM vs. OD_x_) was obtained for each PS. The OD_x_ values were the following: 405 nm for **P1** and **P12**, 410 nm for **P8**, **P10**, **P11**, and **P13**, 415 nm for **P3**, 420 nm for **P2**, **P4**, **P6**, and **P7**, and 435 nm for **P5** and **P9**. The quantity of unbound PSs was inferred by interpolating the data on reference calibration plots. The experimental values were reported as percentages of each PS bound to *S. aureus* cells. The experiments were performed in triplicate.

### 4.7. Photodynamic Treatment of Biofilms

The effect of diaryl-porphyrins in inhibiting the biofilm formation of *S. aureus* was evaluated as follows. The overnight inocula of *S. aureus* ATCC 6538P and ATCC 43300 strains were diluted 500-fold in M9 minimal medium added with glucose (10 mM) and casamino acids (0.2% *w*/*v*). The porphyrins were added at a final concentration of 20 µM and incubated in the dark for 1 h. Upon irradiation with a final dose of 30 J/cm^2^ (100 mW/cm^2^, 300 s), the bacteria were grown overnight at 37 °C to form biofilm. To evaluate the eradication of biofilm, 24-h-old biofilms were treated with PS at a concentration of 30 μM, dark incubated for 1 h, and irradiated (30 J/cm^2^).

In both experimental setups (inhibition of biofilm formation and eradication of formed biofilms), a panel of the following controls was included: DMSO treated and not irradiated biofilm (+DMSO; −light), DMSO treated and irradiated biofilm (+DMSO; +light), PS treated and not irradiated biofilm (+PS; −light), untreated and irradiated biofilm (−PS; +light), and untreated and not irradiated biofilm (−PS; −light).

The adherent biomass was stained with a crystal violet solution (0.1% *w*/*v* for 20 min) upon the removal of planktonic phase and a gentle wash with PBS. The cell viabilities of planktonic (CFU/mL) and adherent (CFU/well) phases were calculated using the colony count method previously reported. The planktonic biomass was collected from the supernatant, while the adherent cells were scraped and suspended in 1 mL of PBS upon removal of the planktonic phase and a single wash with sterile PBS.

### 4.8. Statistical Analyses

The photo-inactivation experiments on suspended cells and biofilm formation by each microbial strain were performed at least three times with independent cultures and the statistical analyses were assessed using one-way ANOVA. If homogeneity of variance was not observed, a post hoc test was performed.

## Figures and Tables

**Table 1:** List of diaryl-porphyrins (**P1**–**P13**) used in this study.

	PS	Chemical Structure	Chemical Denomination	Ref.
**Non-ionic (Ø)**	P1	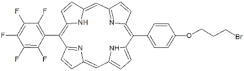	5-Pentafluorophenyl-15-[4-(4-Bromobutoxy)Phenyl]-21H,23H-porphyrin	[23,24]
	P2	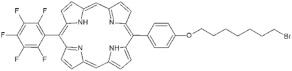	5-Pentafluorophenyl-15-[4-(8-Bromooctaoxy)Phenyl]-21H,23H-porphyrin	[23,24]
	P3	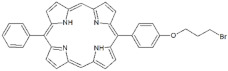	5-Phenyl-15-[4-(4-bromobutoxy)phenyl]-21H,23H-porphyrin	[21]
	P4	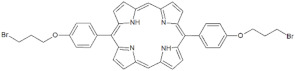	5,15-Di[4-(4-bromobutoxy)phenyl]-21H,23H-porphyrin	[21]
	P5	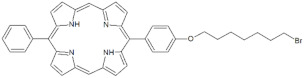	5-Phenyl-15-[4-(8-bromooctanoxy)phenyl]-21H,23H-porphyrin	[21]
	P6	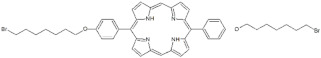	5,15-Di[4-(8-bromooctanoxy)phenyl]-21H,23H-porphyrin	[21]
**Monocationic (+)**	P7	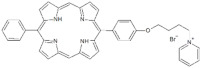	5-Phenyl-15-[4-(4-pyridinobutoxy)phenyl]-21H,23H-porphyrin	[21]
	P8	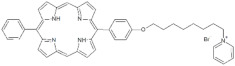	5-Phenyl-15-[4-(4-pyridinooctaoxy)phenyl]-21H,23H-porphyrin	[21]
	P9	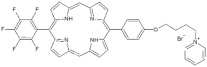	5-Pentafluorophenyl-15-[4-(4-Pyridinobutoxy)Phenyl]-21H,23H-porphyrin	[23]
	P10	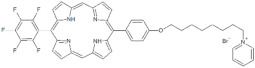	5-Pentafluorophenyl-15-[4-(4-Pyridinooctaoxy)Phenyl]-21H,23H-porphyrin	[23]
**Dicationic (++)**	P11	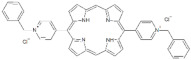	5,15-di(N-benzyl-4-pyridyl)porphyrin	[22,25]
	P12	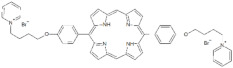	5,15-Di[4-(4-pyridinobutoxy)phenyl]-21H,23H-porphyrin	[21]
	P13	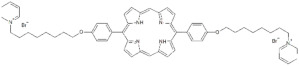	5,15-Di[4-(4-pyridinooctaoxy)phenyl]-21H,23H-porphyrin	[21]

## Data Availability

Not applicable.

## References

[1] Kwiecinski J.M., Horswill A.R. (2020). *Staphylococcus aureus* bloodstream infections: Pathogenesis and regulatory mechanisms. Curr. Opin. Microbiol..

[2] Kluytmans J., Van Belkum A., Verbrugh H. (1997). Nasal carriage of Staphylococcus aureus: Epidemiology, underlying mechanisms, and associated risks. Clin. Microbiol. Rev..

[3] Zheng Y., He L., Asiamah T.K., Otto M. (2018). Colonization of Medical Devices by Staphylococci. Environ. Microbiol..

[4] Marrie T.J., Nelligan J., Costerton J.W. (1982). A scanning and transmission electron microscopic study of an infected endocardial pacemaker lead. Circulation.

[5] Moormeier D.E., Bayles K.W. (2017). *Staphylococcus aureus* biofilm: A complex developmental organism. Mol. Microbiol..

[6] Lindsay J.A., Holden M.T.G. (2004). *Staphylococcus aureus*: Superbug, super genome?. Trends Microbiol..

[7] Pendleton J.N., Gorman S.P., Gilmore B.F. (2013). Clinical relevance of the ESKAPE pathogens. Expert Rev. Anti-Infect. Ther..

[8] De Oliveira D.M.P., Forde B.M., Kidd T.J., Harris P.N.A., Schembri M.A., Beatson S.A., Paterson D.L., Walker M.J. (2020). Antimicrobial resistance in ESKAPE pathogens. Clin. Microbiol. Rev..

[9] Thombre R., Tiwari V., Bhalchandra Patwardhan R., Pardesi K.R., Mulani M.S., Kamble E.E., Kumkar S.N., Tawre M.S. (2019). Emerging Strategies to Combat ESKAPE Pathogens in the Era of Antimicrobial Resistance: A Review. Front. Microbiol..

[10] Berini F., Orlandi V., Gornati R., Bernardini G., Marinelli F. (2022). Nanoantibiotics to fight multidrug resistant infections by Gram-positive bacteria: Hope or reality?. Biotechnol. Adv..

[11] St. Denis T.G., Dai T., Izikson L., Astrakas C., Anderson R.R., Hamblin M.R., Tegos G.P. (2011). All you need is light, antimicrobial photoinactivation as an evolving and emerging discovery strategy against infectious disease. Virulence.

[12] Maisch T. (2020). Photoantimicrobials—An update. Transl. Biophotonics.

[13] Suvorov N., Pogorilyy V., Diachkova E., Vasil’ev Y., Mironov A., Grin M. (2021). Molecular Sciences Derivatives of Natural Chlorophylls as Agents for Antimicrobial Photodynamic Therapy. Int. J. Mol. Sci..

[14] Cieplik F., Deng D., Crielaard W., Buchalla W., Hellwig E., Al-Ahmad A., Maisch T. (2018). Antimicrobial photodynamic therapy—What we know and what we don’t. Crit. Rev. Microbiol..

[15] Soukos N.S., Goodson M. (2011). Photodynamic therapy in the control of oral biofilms. Periodontology.

[16] Ash C., Dubec M., Donne K., Bashford T. (2017). Effect of wavelength and beam width on penetration in light-tissue interaction using computational methods. Lasers Med. Sci..

[17] Wainwright M., Giddens R.M. (2003). Phenothiazinium photosensitisers: Choices in synthesis and application. Dyes Pigments.

[18] Orlandi V.T., Martegani E., Bolognese F., Caruso E. (2022). Searching for antimicrobial photosensitizers among a panel of BODIPYs. Photochem. Photobiol. Sci..

[19] Klausen M., Ucuncu M., Bradley M. (2020). Design of Photosensitizing Agents for Targeted Antimicrobial Photodynamic Therapy. Molecules.

[20] George S., Hamblin M.R., Kishen A. (2009). Uptake pathways of anionic and cationic photosensitizers into bacteria. Photochem. Photobiol. Sci..

[21] Orlandi V.T., Martegani E., Bolognese F., Trivellin N., Garzotto F., Caruso E. (2021). Photoinactivation of *Pseudomonas aeruginosa* Biofilm by Dicationic Diaryl-Porphyrin. Int. J. Mol. Sci..

[22] Orlandi V.T., Martegani E., Bolognese F., Trivellin N., Maťátková O., Paldrychová M., Baj A., Caruso E. (2020). Photodynamic therapy by diaryl-porphyrins to control the growth of *Candida albicans*. Cosmetics.

[23] Caruso E., Malacarne M.C., Banfi S., Gariboldi M.B., Orlandi V.T. (2019). Cationic diarylporphyrins: In vitro versatile anticancer and antibacterial photosensitizers. J. Photochem. Photobiol. B Biol..

[24] Caruso E., Cerbara M., Malacarne M.C., Marras E., Monti E., Gariboldi M.B. (2019). Synthesis and photodynamic activity of novel non-symmetrical diaryl porphyrins against cancer cell lines. J. Photochem. Photobiol. B Biol..

[25] Orlandi V.T., Caruso E., Tettamanti G., Banfi S., Barbieri P. (2013). Photoinduced antibacterial activity of two dicationic 5,15-diarylporphyrins. J. Photochem. Photobiol. B Biol..

[26] Amos-Tautua B.M., Songca S.P., Oluwafemi O.S. (2019). Application of porphyrins in antibacterial photodynamic therapy. Molecules.

[27] Xuan W., Huang L., Wang Y., Hu X., Szewczyk G., Huang Y.-Y., El-Hussein A., Bommer J.C., Nelson M.L., Sarna T. (2019). Amphiphilic tetracationic porphyrins are exceptionally active antimicrobial photosensitizers: In vitro and in vivo studies with the free-base and Pd-chelate. HHS Public Access. J. Biophotonics.

[28] Collins T.L., Markus E.A., Hassett D.J., Robinson J.B. (2010). The Effect of a Cationic Porphyrin on *Pseudomonas aeruginosa* Biofilms. Curr. Microbiol..

[29] Taslı H., Akbıyık A., Topaloğlu N., Alptüzün V., Parlar S. (2018). Photodynamic antimicrobial activity of new porphyrin derivatives against methicillin resistant *Staphylococcus aureus*. J. Microbiol..

[30] Philippova T.O., Galkin B.N., Zinchenko O.Y., Rusakova M.Y., Ivanitsa V.A., Zhilina Z.I., Vodzinskii S.V., Ishkov Y.V. (2003). The antimicrobial properties of new synthetic porphyrins. J. Porphyr. Phthalocyanines.

[31] Burda W.N., Fields K.B., Gill J.B., Burt R., Shepherd M., Zhang X.P., Shaw L.N. (2012). Neutral metallated and meso-substituted porphyrins as antimicrobial agents against Gram-positive pathogens. Eur. J. Clin. Microbiol. Infect. Dis..

[32] Gonzales F.P., Felgenträger A., Bäumler W., Maisch T. (2013). Fungicidal photodynamic effect of a twofold positively charged porphyrin against *Candida albicans* planktonic cells and biofilms. Future Microbiol..

[33] Sobotta L., Skupin-Mrugalska P., Piskorz J., Mielcarek J. (2019). Porphyrinoid photosensitizers mediated photodynamic inactivation against bacteria. Eur. J. Med. Chem..

[34] Alm R.A., Lahiri S.D. (2020). Narrow-Spectrum Antibacterial Agents—Benefits and Challenges. Antibiotics.

[35] Ghorbani J., Rahban D., Aghamiri S., Teymouri A., Bahador A. (2018). Photosensitizers in antibacterial photodynamic therapy: An overview. Laser Ther..

[36] Le K.Y., Otto M. (2015). Quorum-sensing regulation in staphylococci-an overview. Front. Microbiol..

[37] Jenul C., Horswill A.R. (2018). Regulation of *Staphylococcus aureus* Virulence. Microbiol. Spectr..

[38] De I., Ribeiro P., Guerra Pinto J., Müller B., Souza N., Miñán A.G., Ferreira-Strixino J. (2022). Antimicrobial photodynamic therapy with curcumin on methicillin-resistant *Staphylococcus aureus* biofilm. Photodiagnosis Photodyn. Ther..

[39] Beirão S., Fernandes S., Coelho J., Faustino M.A.F., Tomé J.P.C., Neves M.G.P.M.S., Tomé A.C., Almeida A., Cunha A. (2014). Photodynamic Inactivation of Bacterial and Yeast Biofilms with a Cationic Porphyrin. Photochem. Photobiol..

[40] Mamone L., Ferreyra D.D., Gándara L., Di Venosa G., Vallecorsa P., Sáenz D., Calvo G., Batlle A., Buzzola F., Durantini E.N. (2016). Photodynamic inactivation of planktonic and biofilm growing bacteria mediated by a meso-substituted porphyrin bearing four basic amino groups. J. Photochem. Photobiol. B Biol..

[41] Orlandi V.T., Martegani E., Bolognese F. (2018). Catalase A is involved in the response to photooxidative stress in *Pseudomonas aeruginosa*. Photodiagnosis Photodyn. Ther..

